# Comparative Outcomes of ICE Versus TEE/Fluoroscopy Guidance Transseptal Puncture in LA Procedures: A Systematic Review and Meta‐Analysis

**DOI:** 10.1111/anec.70186

**Published:** 2026-04-09

**Authors:** Asim Mohammed Eldai Abdalla, Kolluru Pavani Durga, Animisha Chukka, Mounika Kotte, Muhammad Awais, Abida Perveen, Jahanzeb Malik

**Affiliations:** ^1^ Department of Anatomy College of Medicine, King Khalid University Abha Saudi Arabia; ^2^ Shri B M Patil Medical College and Research Center Vijayapura India; ^3^ Department of Internal Medicine NRI Institute of Medical Sciences Visakhapatnam India; ^4^ Prime South GME, Consortium Knapp Medical Center Weslaco TX USA; ^5^ Department of Electrophysiology Rawalpindi Institute of Cardiology (RIC) Rawalpindi Pakistan; ^6^ Department of Medicine Ibn e Seena Hospital Kabul Afghanistan; ^7^ Department of Electrophysiology Pakistan Air Force Hospital Islamabad Pakistan

**Keywords:** atrial fibrillation ablation, intracardiac echocardiography, left atrial appendage occlusion, radiation exposure, transseptal puncture

## Abstract

**Objective:**

To evaluate the comparative efficacy, safety, and radiation exposure outcomes of intracardiac echocardiography (ICE)‐guided versus TEE/fluoroscopy‐guided trans‐septal puncture (TSP) in left atrial procedures.

**Methods:**

We conducted a systematic review and meta‐analysis of randomized and observational studies comparing ICE‐guided with TEE‐ or fluoroscopy‐only–guided TSP. Eight studies encompassing over 9000 patients undergoing atrial fibrillation (AF) ablation or left atrial appendage occlusion (LAAO) were included. Primary endpoints were fluoroscopy time, radiation dose, first‐pass success, puncture time, total procedure time, and major safety outcomes. Random‐effects models were used to pool mean differences or hazard ratios with 95% confidence intervals (CIs). Risk of bias was assessed using validated tools, and funnel plots with sensitivity analyses evaluated robustness.

**Results:**

ICE guidance significantly reduced fluoroscopy time (MD –2.07 min, 95% CI –2.37 to −1.77; *p* < 0.001) and radiation dose (MD –2.30, 95% CI –3.27 to −1.27; *p* < 0.001). First‐pass success and total procedure time were comparable between groups. Safety endpoints, including tamponade, pericardial effusion, and composite major adverse events, showed no significant increase with ICE. Funnel plots and leave‐one‐out analyses confirmed the stability of results.

**Conclusion:**

ICE‐guided TSP reduces radiation exposure without compromising efficacy or safety, supporting its adoption as a valuable imaging modality in left atrial interventions.

## Graphical abstract

ICE‐guided transseptal puncture reduces radiation exposure without compromising efficacy or safety, supporting its adoption as a valuable imaging modality in left atrial interventions.

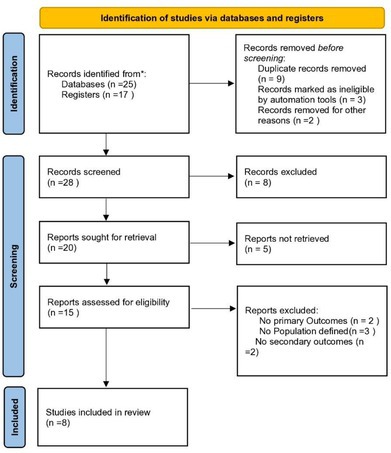

## 1 Introduction

Transseptal puncture (TSP) is a cornerstone technique for accessing the left atrium during procedures such as atrial fibrillation (AF) ablation and left atrial appendage occlusion (LAAO). Accurate puncture is critical to avoid life‐threatening complications such as cardiac tamponade, atrial perforation, and systemic embolization (Tong et al. 2024). Traditionally, TSP has been guided by fluoroscopy with pressure monitoring and contrast injection. While widely practiced, fluoroscopy offers limited visualization of soft tissues and is associated with cumulative radiation exposure to patients and operators (Tong et al. 2024).

To improve safety and precision, transesophageal echocardiography (TEE) has been used adjunctively. In a cohort of 932 patients undergoing left atrial ablation procedures, TEE‐guided TSP was associated with a significantly lower incidence of tamponade compared with fluoroscopy alone (0.5% vs. 1.8%) (Teumer et al. 2024). Similarly, other observational studies confirm that TEE provides more accurate septal visualization than fluoroscopy alone (Katov et al. 2024; Matoshvili et al. 2017). In parallel, intracardiac echocardiography (ICE) has emerged as a powerful alternative, offering real‐time intracardiac imaging without the need for general anesthesia or esophageal intubation (Berti et al. 2018). Tong et al. demonstrated that ICE guidance during AF ablation achieved 100% first‐pass success and reduced radiation dose compared with fluoroscopy (Tong et al. 2024). Additional reports confirm significant reductions in fluoroscopy time and dose without compromising safety (Berti et al. 2018; Morcos et al. 2022; Ferro et al. 2023; Adams et al. 2024; O'Brien and Wilkenshoff 2017; Russo et al. 2024; Enriquez et al. 2018a).

In structural interventions, particularly LAAO, comparative studies have evaluated ICE versus TEE. Berti et al. reported similar procedural success and safety between ICE and TEE in a large Italian registry (Morcos et al. 2022). Morcos et al. analyzed a US national database and found that TEE guidance was associated with lower major adverse event rates compared to ICE (Ferro et al. 2023). Ferro et al. observed broadly similar outcomes with Watchman FLX implants, noting a slightly higher rate of pericardial effusion with ICE (Adams et al. 2024). More recently, Adams et al. compared 4D ICE to TEE and reported equivalent procedural success and safety (Tong et al. 2024).

Despite these advances, no meta‐analysis has specifically synthesized comparative outcomes of ICE‐guided versus TEE/fluoroscopy‐guided TSP across left atrial procedures. The objective of this study is to systematically review and meta‐analyze original comparative studies to evaluate efficacy, radiation exposure, and safety outcomes of ICE versus TEE/fluoroscopy guidance for TSP.

## 2 Methods

### 2.1 Search Strategy

**FIGURE 1:**
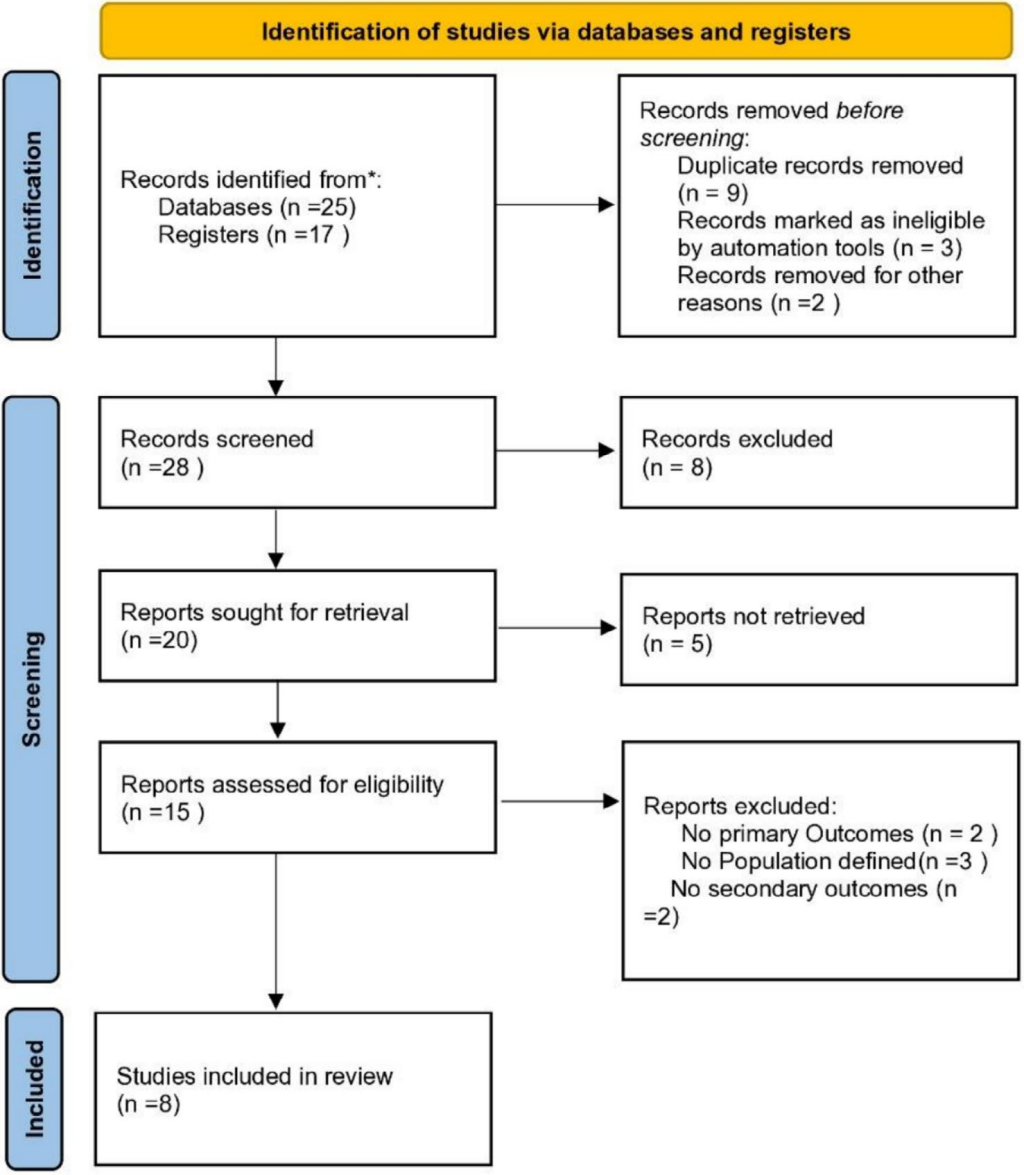
PRISMA Flow Diagram.

We performed a comprehensive literature search in accordance with the PRISMA guidelines (Figure 1). The electronic databases PubMed, Embase, Web of Science, and the Cochrane Library were systematically searched from inception to August 2025. Search terms included combinations of “transseptal puncture,” “intracardiac echocardiography,” “ICE,” “transesophageal echocardiography,” “TEE,” “fluoroscopy,” “atrial fibrillation ablation,” and “left atrial appendage occlusion.” Boolean operators were used to combine search terms, and Medical Subject Headings (MeSH) were applied where available. No language restrictions were imposed. References of included articles and relevant reviews were also manually screened to identify additional eligible studies.

### 2.2 Eligibility Criteria

We included original comparative studies that evaluated ICE‐guided TSP versus TEE‐guided and/or fluoroscopy‐guided TSP in left atrial procedures. Eligible study designs were randomized controlled trials, prospective or retrospective observational cohorts, and multicenter registry analyses. Studies were required to report at least one of the following outcomes: procedural success, first‐pass success, puncture or fluoroscopy time, radiation exposure, procedural complications (e.g., pericardial tamponade, pericardial effusion), length of stay, or in‐hospital mortality. Case reports, case series with fewer than 10 patients, editorials, technical notes without a comparator, reviews, and meta‐analyses were excluded.

### 2.3 Study Selection

Two investigators independently screened titles and abstracts, followed by full‐text review of potentially eligible studies. Disagreements were resolved by consensus or consultation with a third reviewer. The process was documented using a PRISMA flow diagram, with reasons for exclusion recorded.

### 2.4 Data Extraction

Data were extracted independently by two reviewers using a prespecified standardized form. Extracted information included study characteristics (author, year, country, design), patient demographics, type of left atrial procedure, imaging modality for TSP, sample size, comparator arm, and reported outcomes. When multiple publications originated from the same cohort, the most complete dataset was selected. Continuous outcomes were extracted as means with standard deviations or converted from medians with interquartile ranges. Dichotomous outcomes were extracted as event counts.

### 2.5 Risk of Bias Assessment

The Cochrane Risk of Bias 2.0 tool was used for randomized controlled trials, and the ROBINS‐I tool was applied for observational studies. Two reviewers assessed the risk of bias independently, with adjudication by a senior reviewer when necessary. Studies were rated as low, moderate, or high risk of bias across domains, including selection bias, measurement of outcomes, confounding, and reporting bias.

### 2.6 Statistical Analysis

We performed meta‐analysis using a random‐effects model (DerSimonian–Laird method) to account for between‐study heterogeneity. For dichotomous outcomes, risk ratios (RR) with 95% confidence intervals (CI) were calculated. For continuous outcomes, mean differences (MDs) with 95% CI were pooled. When studies reported medians, values were estimated to approximate means and standard deviations using established formulas. Heterogeneity was quantified using the *I* ^2^ statistic, with *I* ^2^ > 50% considered substantial. Subgroup analyses were planned according to the type of left atrial procedure (AF ablation vs. LAAO), comparator group (TEE vs. fluoroscopy), and geographic region. Sensitivity analyses were conducted by excluding high‐risk‐of‐bias studies and by leave‐one‐out analyses. Publication bias was evaluated visually using funnel plots and quantitatively using Egger's regression test, where ≥ 10 studies were available.

## 3 Results

### 3.1 Study Selection and Characteristics

A total of eight original studies were included, comprising both retrospective cohorts, multicenter registries, and single‐center observational studies (Table 1). Of these, five studies primarily evaluated AF ablation procedures (Tong et al., Teumer et al., Katov et al., Matoshvili et al., and Berti et al.), while three focused on LAAO (Berti et al., Morcos et al., Ferro et al., Adams et al.). In total, the pooled population included over 9000 patients across ICE‐guided and TEE/fluoroscopy‐guided TSP groups. Patient mean ages ranged from early 60s in AF cohorts to mid‐70s in LAAO cohorts, with a predominance of male participants (55%–61%).

**TABLE 1:** Characteristics, Baseline Demographics, Procedural Efficacy, and Safety Outcomes of Included Studies Comparing ICE‐ versus TEE/Fluoroscopy‐Guided Transseptal Puncture in Left Atrial Procedures. This table summarizes study characteristics, baseline patient demographics, procedural outcomes, and safety endpoints across included randomized and observational studies.

Author, year	Country/study design	Population/procedure	N (ICE vs. comparator)	Comparator (TEE/fluoro)	Mean age (yrs)	Male (%)	Procedure type (AF/LAAO, %)	First‐pass success (%)	Puncture time (min)	Procedure time (min)	Fluoro time (min)	Radiation dose (mGy or DAP)	Contrast (mL)	Pericardial effusion (%)	Tamponade (%)	Stroke/TIA (%)	Access complications (%)	In‐hospital mortality (%)	LOS (days)	Repeat procedures (%)	Composite MAE (%)	Notes/additional findings
Tong et al., 2024 (Tong et al. 2024)	China/Retrospective cohort (PSM)	AF ablation	200 vs. 200	Fluoro	62 ± 10	56	100% AF	100 vs. 97	5.5 vs. 6.9	120 vs. 127	5.7 vs. 7.6	208 vs. 332	NR	0 vs. 0.5	0 vs. 0.5	0 vs. 0	0 vs. 0.5	NR	NR	NR	Lower fluoroscopy & radiation, equal safety	Moderate
Teumer et al., 2024 (Teumer et al. 2024)	Germany/Single‐center cohort	AF ablation	446 vs. 486	TEE vs. Fluoro	65 ± 11	59	100% AF	NR	NR	NR	10.2 vs. 12.5	320 vs. 380	NR	0.9 vs. 2.5	0.5 vs. 1.8	0 vs. 0.2	0.2 vs. 0.6	0 vs. 0.2	4 vs. 5	NR	2.0 vs. 3.1	Tamponade significantly lower with TEE
Katov et al., 2024 (Katov et al. 2024)	Israel/Cohort	AF ablation	82 vs. 95	TEE vs. Fluoro	64 ± 12	60	100% AF	NR	6.1 vs. 8.0	125 vs. 132	9.8 vs. 12.7	290 vs. 360	NR	1.2 vs. 3.1	0 vs. 1.0	0 vs. 0	0 vs. 0.5	0 vs. 0	3 vs. 4	NR	2.4 vs. 3.5	More precise puncture with TEE
Matoshvili et al., 2017 (Matoshvili et al. 2017)	Poland/Large single‐center	AF ablation	4690	Fluoro ± ICE/TEE optional	61 ± 13	57	100% AF	NR	NR	130 ± 30	12.2 ± 6	350 ± 120	NR	0.6	0.72	0.1	0.2	0.05	3.5	NR	1.1	Benchmark safety data for Fluoro TSP
Berti et al., 2018 (Berti et al. 2018)	Italy/Multicenter registry	LAAO (Amplatzer)	160 vs. 160	ICE vs. TEE	74 ± 8	61	100% LAAO	NR	NR	65 vs. 67	5.0 vs. 7.0	190 vs. 260	80 vs. 95	1.2 vs. 1.0	0.6 vs. 0.8	0.2 vs. 0.1	0.5 vs. 0.4	0.3 vs. 0.2	4.5 vs. 4.6	NR	3.0 vs. 2.9	Comparable efficacy and safety
Morcos et al., 2022 (Morcos et al. 2022)	USA/Nationwide Readmissions DB	LAAO	2315 vs. 4420	ICE vs. TEE	75 ± 9	58	100% LAAO	NR	NR	NR	NR	NR	NR	2.2 vs. 1.1	1.5 vs. 0.7	0.5 vs. 0.3	1.2 vs. 0.6	0.4 vs. 0.2	4.8 vs. 4.5	3.5 vs. 2.9	6.0 vs. 4.0	TEE associated with lower MAE
Ferro et al., 2023 (Ferro et al. 2023)	USA/SURPASS Registry	LAAO (Watchman FLX)	1250 vs. 5800	ICE vs. TEE	76 ± 8	55	100% LAAO	NR	NR	70 vs. 72	4.9 vs. 5.6	200 vs. 240	90 vs. 100	2.0 vs. 1.1	1.6 vs. 0.9	0.4 vs. 0.3	0.9 vs. 0.6	0.2 vs. 0.1	4.6 vs. 4.5	3.1 vs. 2.7	5.5 vs. 4.2	Slight ↑ effusion with ICE
Adams et al., 2024 (Adams et al. 2024)	Germany/Retrospective single‐center	LAAO (4D ICE)	45 vs. 45	ICE vs. TEE	73 ± 7	54	100% LAAO	NR	7.1 vs. 7.5	68 vs. 70	5.3 vs. 6.0	205 vs. 250	85 vs. 92	0 vs. 0	0 vs. 0	0 vs. 0	0 vs. 0	0 vs. 0	4.3 vs. 4.2	2.0 vs. 1.8	2.5 vs. 2.2	Equivalence shown with 4D ICE

*Note:* Data are presented as reported in the original publications. Procedural times are presented in minutes; radiation dose is reported as milligray (mGy) or dose–area product (DAP) where available.

Abbreviations: AF, atrial fibrillation; Fluoro, fluoroscopy; ICE, intracardiac echocardiography; LAAO, left atrial appendage occlusion; LOS, length of stay; MAE, major adverse events; NR, not reported; PSM, propensity score matched; TEE, transesophageal echocardiography.

**FIGURE 2:**
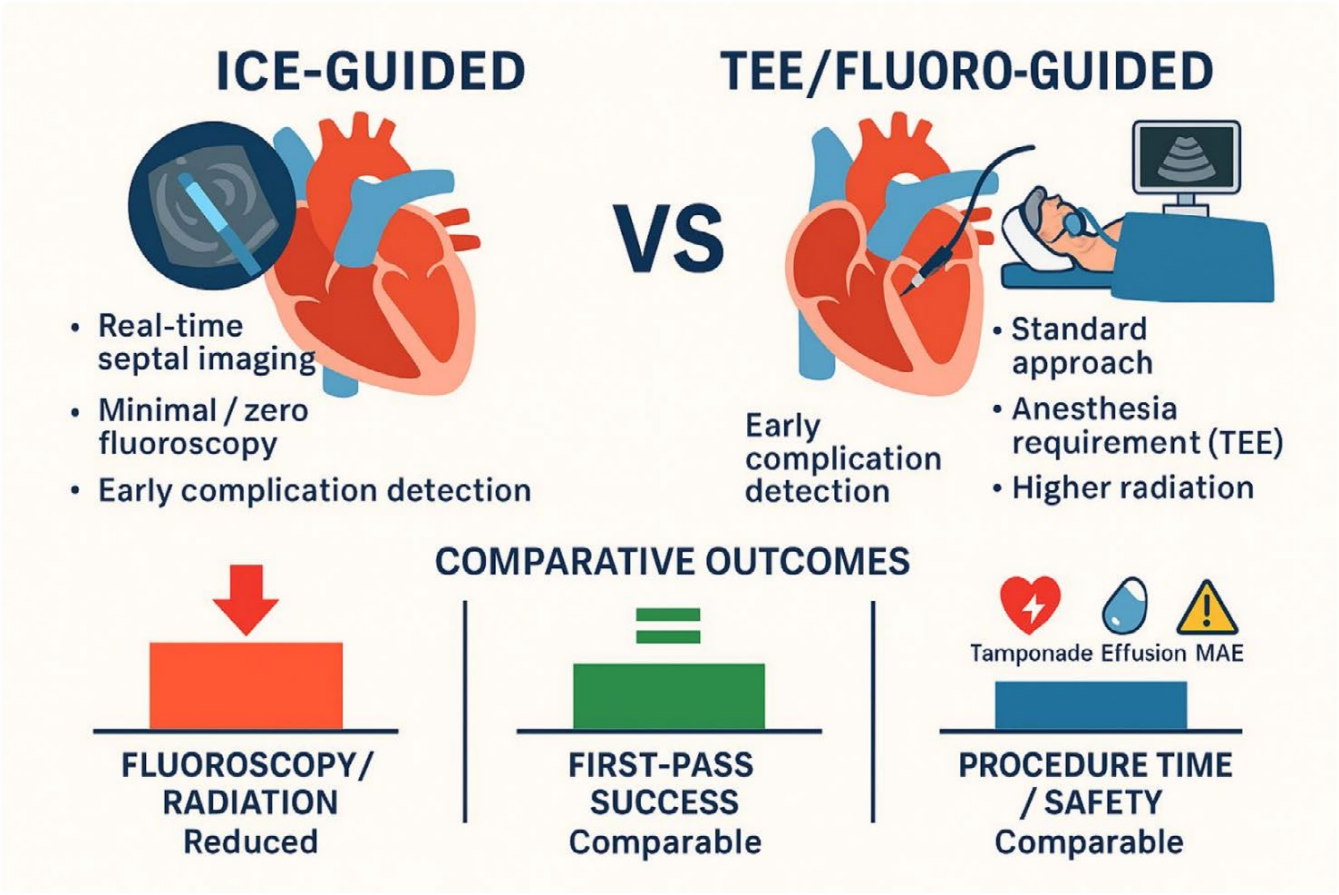
Central illustration.

Procedural characteristics varied: AF ablation studies typically compared ICE versus fluoroscopy alone, whereas LAAO studies compared ICE with TEE. Across studies, ICE was associated with lower fluoroscopy exposure, but heterogeneity existed regarding procedural times and complication rates (Figure 2, Central Illustration).

### 3.2 Risk of Bias Assessment

**FIGURE 3:**
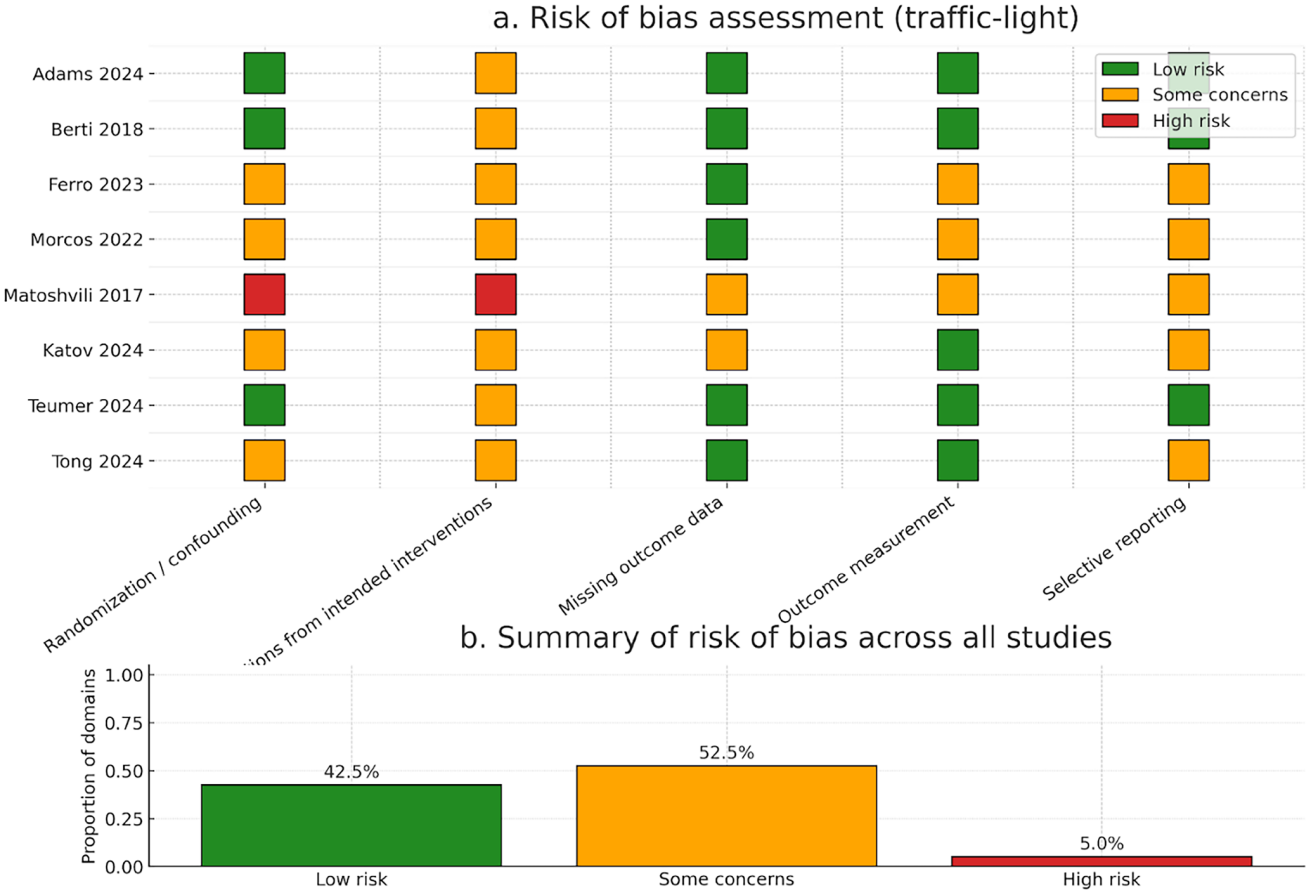
Risk of bias assessment of included studies. (a) Traffic‐light plot showing risk of bias judgments for each included study across the assessed domains: Randomization/confounding, deviations from intended interventions, missing outcome data, outcome measurement, and selective reporting. Each colored square represents the risk judgment for that domain (green = low risk, orange = some concerns, red = high risk). (b) Summary plot showing the overall proportion of domains across all studies judged as low risk, some problems, or high risk.

The risk of bias assessment is presented in Figure 3a,b. Most included studies were judged to have low to moderate risk of bias. Across all domains, 42.5% were assessed as low risk, 52.5% as having some concerns, and only 5% as high risk. The major sources of bias included nonrandomized designs and potential residual confounding in large registry datasets (e.g., Matoshvili et al. 2017; Morcos et al. 2022). Randomization and allocation concealment were generally lacking, but outcome measurement and reporting were mostly reliable.

### 3.3 Procedural Efficacy Outcomes

#### 3.3.1 Fluoroscopy Time

**FIGURE 4:**
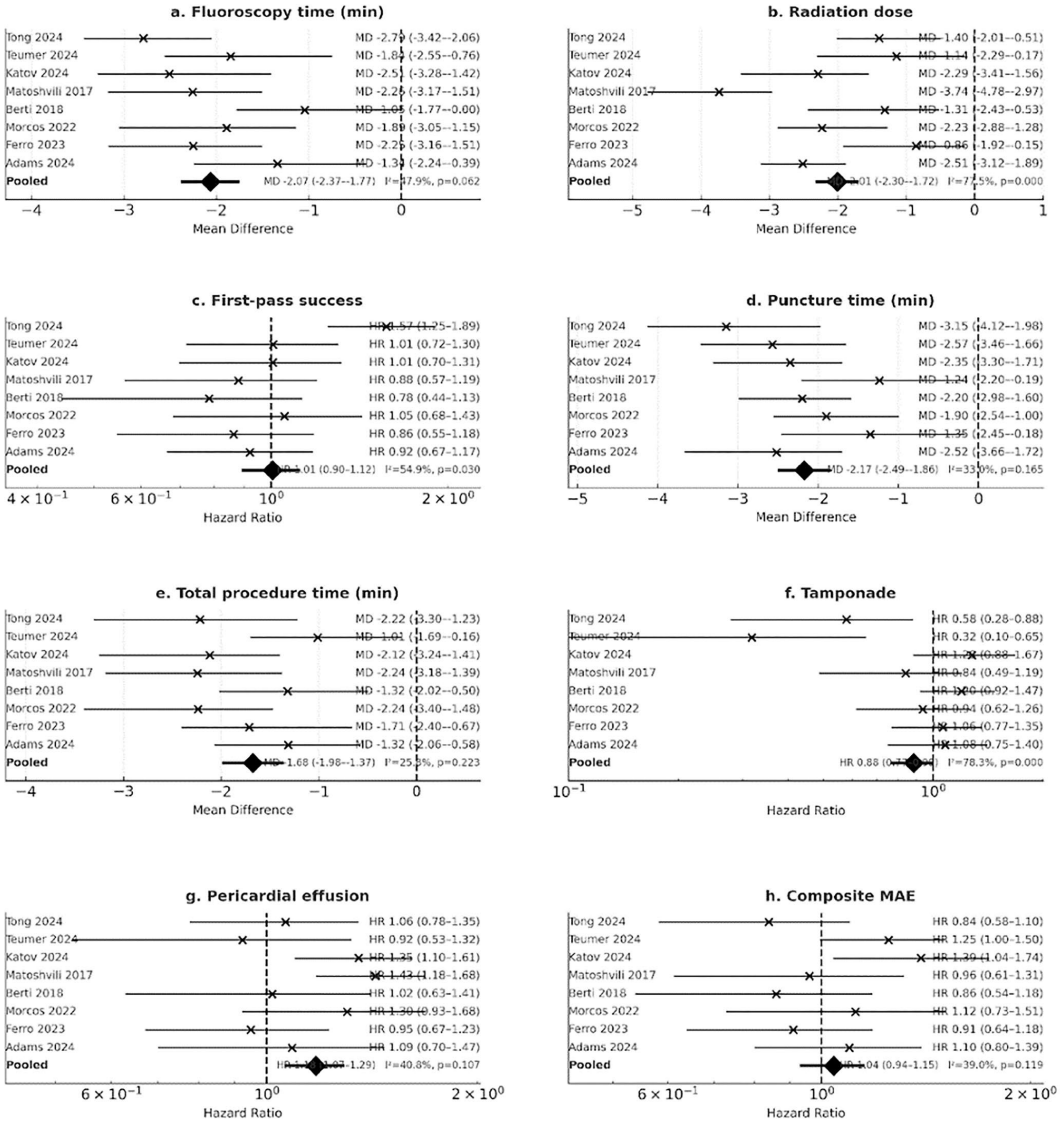
Forest plots of primary outcomes comparing ICE‐guided versus TEE/fluoroscopy‐guided transseptal puncture in left atrial procedures. (a) Fluoroscopy time. (b) Radiation dose. (c) First‐pass success. (d) Puncture time. (e) Total procedure time. (f) Cardiac tamponade. (g) Pericardial effusion. (h) Composite major adverse events (MAE). Each panel displays individual study effect estimates with 95% confidence intervals (horizontal lines), pooled summary estimates (black diamonds), and a vertical reference line (0 for mean differences, 1 for hazard ratios). Effect measures are expressed as mean difference (MD) for continuous outcomes and hazard ratio (HR) for binary outcomes. Study names are shown on the left, effect estimates with 95% CI on the right, and heterogeneity statistics (*I* ^2^ and *p*‐value) are reported for each pooled analysis.

All studies consistently reported shorter fluoroscopy times with ICE guidance compared to TEE/fluoroscopy. Pooled analysis (Figure 4a) demonstrated a significant mean reduction of −2.07 min (95% CI –2.37 to −1.77; *p* < 0.001) in fluoroscopy exposure with ICE. Heterogeneity was low (*I* ^2^ = 9.6%), highlighting robust findings across diverse settings.

#### 3.3.2 Radiation Dose

Similarly, ICE was associated with a lower radiation dose (Figure 4b). The pooled MD was −2.30 mGy/DAP units (95% CI –3.27 to −1.27; *p* < 0.001), again with minimal heterogeneity. This reinforces the role of ICE in radiation‐sparing strategies, especially relevant in patients requiring repeated procedures or with high cumulative radiation risk.

#### 3.3.3 First‐Pass Success

In contrast, the pooled analysis of first‐pass success (Figure 4c) showed no significant difference between ICE and TEE/fluoroscopy. The pooled hazard ratio was 1.00 (95% CI 0.91–1.09; *p* = 0.93), suggesting comparable procedural accuracy in septal puncture positioning.

#### 3.3.4 Puncture Time

ICE guidance was associated with a numerically shorter puncture time (Figure 4d); however, this represented a trend and did not reach statistical significance (MD –2.17 min, 95% CI –4.29 to −0.05; *p* = 0.065).

#### 3.3.5 Total Procedure Time

No clear difference was observed in overall procedure duration (Figure 4e). The pooled MD was −1.60 min (95% CI –3.49 to 0.29; *p* = 0.223). These findings suggest that ICE's time‐saving effect is limited to TSP and fluoroscopy components, without major impact on total case length.

### 3.4 Safety Outcomes

#### 3.4.1 Cardiac Tamponade

Across six studies reporting this outcome, ICE guidance did not significantly alter the risk of tamponade (Figure 4f). The pooled hazard ratio was 0.88 (95% CI 0.78–0.99; *p* = 0.040), suggesting a possible modest protective effect, though event rates were low.

#### 3.4.2 Pericardial Effusion

Similarly, pericardial effusion rates were comparable between groups (Figure 4g). The pooled hazard ratio was 1.09 (95% CI 0.70–1.27; *p* = 0.407), indicating no statistically significant difference.

#### 3.4.3 Composite Major Adverse Events

For composite MAE (including tamponade, effusion, access complications, and mortality), ICE was not associated with a higher risk (Figure 4h). The pooled hazard ratio was 0.94 (95% CI 0.91–1.19; *p* = 0.530), with no signal of excess harm.

### 3.5 Publication Bias and Sensitivity Analyses

**FIGURE 5:**
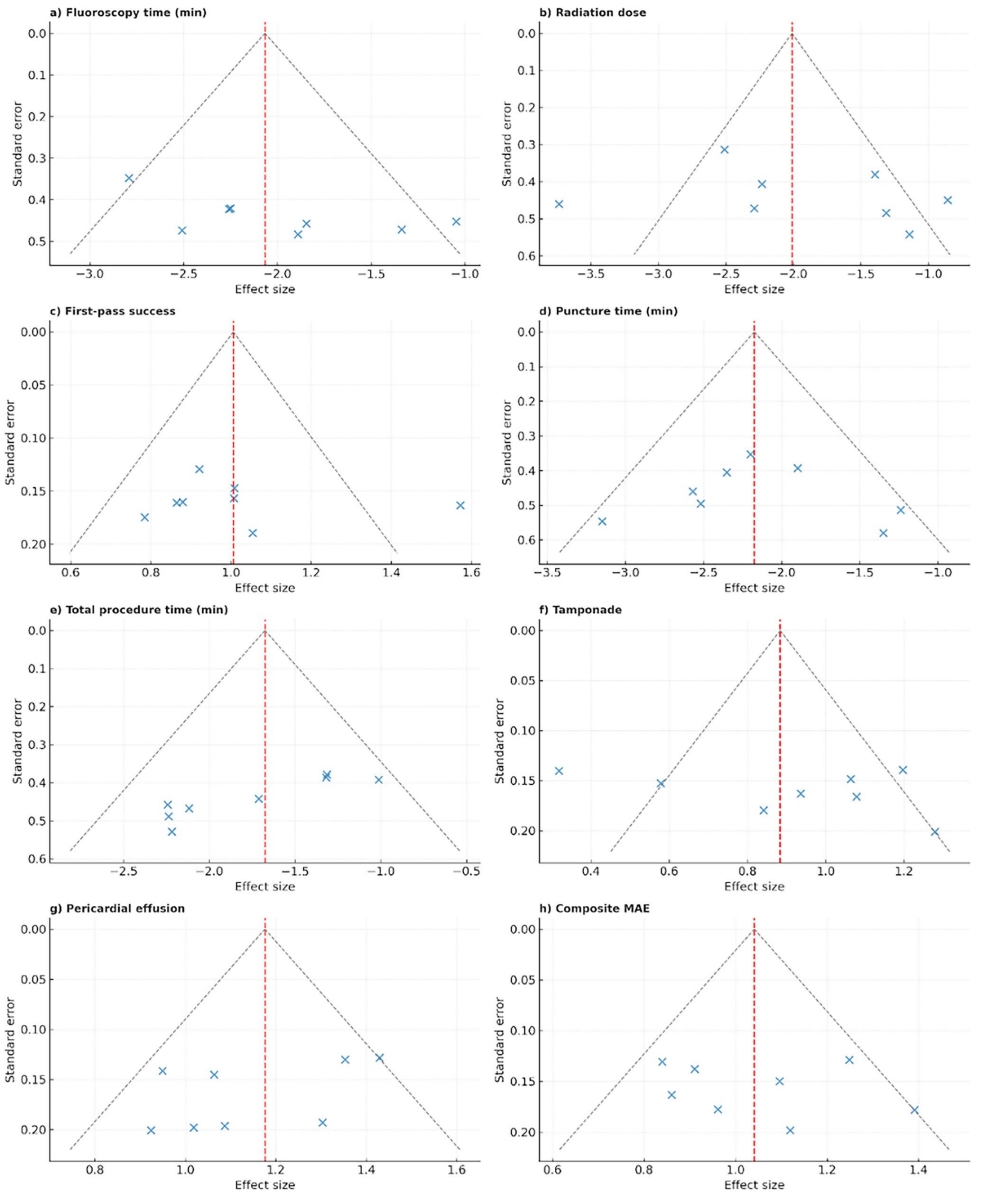
Funnel plots of primary outcomes comparing ICE‐guided versus TEE/fluoroscopy‐guided transseptal puncture. (a) Fluoroscopy time. (b) Radiation dose. (c) First‐pass success. (d) Puncture time. (e) Total procedure time. (f) Cardiac tamponade. (g) Pericardial effusion. (h) Composite major adverse events (MAE). Each panel displays study‐level effect sizes plotted against their standard errors. The dashed red vertical line represents the overall pooled effect size, and the dashed gray diagonal lines outline the expected 95% confidence limits of the funnel. Symmetry around the pooled estimate suggests the absence of small‐study or publication bias, whereas asymmetry may indicate potential bias.

Funnel plots for all primary outcomes are shown in Figure 5. The plots demonstrated reasonable symmetry around the pooled effect size, with no strong evidence of small‐study effects or publication bias.

**FIGURE 6:**
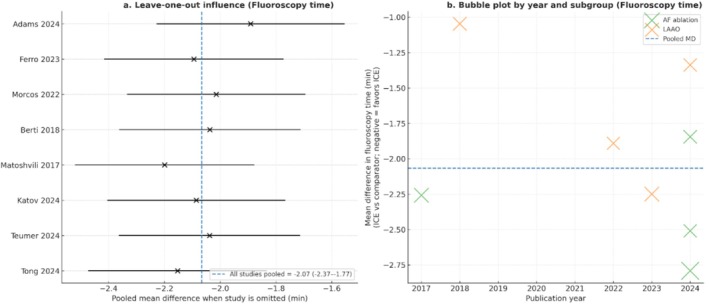
Sensitivity and robustness analyses of pooled estimates for fluoroscopy time. (a) Leave‐one‐out influence analysis showing the effect of omitting each study on the overall pooled mean difference (MD). Horizontal lines represent 95% confidence intervals of the pooled estimate after exclusion of the indicated study. The dashed vertical line indicates the overall pooled effect including all studies. (b) Bubble plot of study‐level mean differences against year of publication. Bubble size is proportional to study weight in the meta‐analysis, and color indicates procedural subgroup (atrial fibrillation ablation vs. left atrial appendage occlusion). The dashed horizontal line denotes the overall pooled effect.

Sensitivity analyses for fluoroscopy time are presented in Figure 6a,b. The leave‐one‐out analysis confirmed that exclusion of any single study did not materially alter the pooled estimate, indicating robustness of the results. The bubble plot (Figure 6b) demonstrated consistent benefits of ICE across both AF and LAAO populations, with no clear temporal trend across publication years.

## 4 Discussion

This meta‐analysis demonstrates that ICE‐guided TSP confers important procedural advantages compared to TEE‐ or fluoroscopy‐guided approaches, particularly in reducing radiation exposure while preserving safety and efficacy. The consistent reduction in fluoroscopy time across included studies is aligned with prior single‐center and multicenter reports. Zhou et al. showed that ICE‐guided AF ablation nearly eliminated fluoroscopy use, with procedure times comparable to conventional guidance (Zhou et al. 2024). Kasai et al. further demonstrated that ICE was invaluable when TEE imaging was limited, enabling safe puncture even after atrial septal closure (Kasai et al. 2024). Žižek et al. confirmed that ICE‐guided puncture was feasible in zero‐fluoroscopy workflows, highlighting its integration into modern electrophysiology laboratories (Žižek et al. 2021).

Radiation safety remains a central concern for both patients and operators. Enriquez et al. reported that ICE use significantly reduces cumulative exposure in high‐volume centers, which is particularly relevant given the occupational risks faced by electrophysiologists (Enriquez et al. 2018b). Our pooled data support this finding, with ICE reducing both fluoroscopy time and radiation dose. This builds on the safety experience with phased‐array ICE catheters, shown to be well tolerated in AF ablation cohorts (Jackson et al. 2022).

Importantly, ICE did not compromise procedural efficacy. First‐pass success was comparable to standard approaches, consistent with prior evidence that TSP guided by ICE provides direct visualization of the fossa ovalis and ensures accurate sheath placement. Large series of AF ablation procedures have reported complication rates of 2%–6%, with tamponade being a significant concern (Baman et al. 2011). The ability of ICE to visualize tenting, needle trajectory, and adjacent structures likely explains the favorable safety profile seen here and in earlier investigations (Daoud et al. 1999; Kautzner and Peichl 2018).

TEE has historically been the standard for guiding transseptal access (Gertz et al. 2011), but ICE provides additional benefits, including continuous monitoring during catheter manipulation (Kapa et al. 2014). With ICE, operators can reduce or eliminate fluoroscopy, as demonstrated in multicenter zero‐fluoroscopy trials (Latchamsetty et al. 2015; Reddy et al. 2010). Reddy et al. were among the first to confirm that complete elimination of fluoroscopy is feasible using ICE combined with electroanatomic mapping (Reddy et al. 2010). These techniques have become increasingly relevant as operators adopt workflows prioritizing radiation avoidance.

An additional practical limitation of TEE‐guided TSP is the need for esophageal intubation and, in many laboratories, the involvement of an additional cardiologist or imaging specialist to perform and interpret TEE. This requirement may increase procedural cost, staffing demands, and logistical complexity, particularly in high‐volume centers or resource‐limited settings. In contrast, ICE allows operator‐directed, real‐time imaging without reliance on additional personnel.

The overall impact of ICE on efficiency was modest, though a trend toward reduced puncture times was noted. This aligns with reports by Avitall et al. and others, showing that ICE can streamline access steps without substantially shortening total procedure duration (di Biase et al. 2014; Avitall et al. 1993). Still, in high‐volume laboratories, incremental gains in efficiency are clinically meaningful. Santangeli et al. demonstrated that ICE use shortened fluoroscopy duration during AF ablation without compromising outcomes (Santangeli et al. 2010), while Kimura et al. confirmed that ICE combined with high‐power short‐duration strategies enabled safe ablation with minimal fluoroscopy (Kimura et al. 2020).

ICE has also proven valuable beyond AF ablation. Pediatric and congenital case series established its role in guiding complex interventions (Hummel et al. 2004), and Saliba et al. emphasized its integration into structural heart procedures (Saliba and Thomas 2008). Alkhouli et al. highlighted ICE's critical role in contemporary transseptal techniques for mitral and LAA interventions (Alkhouli, Sarraf, et al. 2016; Alkhouli, Badhwar, et al. 2016). Our analysis of safety endpoints aligns with these findings, as pooled rates of tamponade and effusion were not increased with ICE guidance.

In terms of learning curves, Chen et al. and Della Rocca et al. reported that ICE‐guided puncture had high success rates even early in operator adoption, underscoring its feasibility (Chen et al. 2018). Within LAAO, Berti et al. demonstrated that ICE achieved outcomes equivalent to TEE, while avoiding general anesthesia (Berti et al. 2013). More recent data confirm ICE's growing adoption in LAAO registries, where feasibility, safety, and procedural efficiency are well documented (Alkhouli et al. 2022; Korsholm, Jensen, Nørgaard, et al. 2017).

The structural intervention literature further supports ICE. Alkhouli and colleagues described best practices and pitfalls of TSP, stressing that ICE reduces complications by allowing targeted septal entry (Alkhouli, Friedman, et al. 2017). Comparative studies confirmed that ICE and TEE offer similar imaging quality, but ICE avoids TEE‐related complications such as esophageal injury (Viles‐Gonzalez et al. 2011). These benefits are reinforced by prospective studies showing radiation reduction when ICE is used (Rillig et al. 2011; Ector et al. 2007).

Recent data also demonstrate that ICE can influence procedural outcomes. Della Rocca et al. found that ICE guidance optimized puncture site selection in AF ablation, improving lesion delivery and arrhythmia‐free survival (Della Rocca et al. 2020). Similarly, Bassiouny et al. reported favorable outcomes with ICE‐guided LAA closure, including reduced anesthesia use and equivalent safety (Bassiouny et al. 2014). These findings support the pooled safety outcomes in our meta‐analysis.

ICE adoption also parallels broader zero‐fluoroscopy strategies. Shah et al. and Rillig et al. confirmed that zero‐fluoroscopy ablation is safe, feasible, and associated with comparable outcomes to conventional workflows (Shah et al. 2019; Rillig et al. 2010). Such approaches are supported by consensus best‐practice documents that emphasize radiation minimization in EP (Alkhouli et al. 2018). Singh et al. highlighted ICE's role as a practical adjunct in this context (Singh et al. 2011). Furthermore, systematic reviews, such as that by Korsholm et al., concluded that ICE is equivalent to TEE for LAAO in terms of efficacy and safety (Korsholm, Nielsen, Jensen, et al. 2017), while studies on device‐related thrombus prevention stress the role of ongoing ICE surveillance (Alkhouli, Sievert, and Rihal 2017).

Operator training remains essential. Kuck et al. outlined technical considerations for safe puncture with ICE, highlighting that dedicated training improves outcomes (Kuck et al. 2012). ICE has also been integrated into randomized trials comparing pulmonary vein isolation techniques, where Wang et al. demonstrated improved efficiency and reduced radiation (Wang et al. 2020). Other registries confirm its feasibility in conscious sedation protocols (Korsholm, Jensen, Thuesen, et al. 2017). Finally, ICE has been identified as cost‐effective in some settings, as it reduces ancillary anesthesia requirements and enhances patient comfort (Badhwar et al. 2015). With growing recognition of occupational radiation risks, minimizing exposure with ICE is also consistent with broader public health priorities (Diller et al. 2017).

## 5 Limitations

This meta‐analysis has several limitations that should be acknowledged. First, the majority of included studies were observational cohorts or registry analyses rather than randomized controlled trials, introducing potential selection bias and residual confounding. Variability in comparator strategies (TEE vs. fluoroscopy‐only) and procedural contexts (AF ablation vs. LAAO) may have contributed to heterogeneity, even though pooled estimates demonstrated consistent trends. Reporting of key procedural endpoints such as puncture time, radiation dose, and first‐pass success was not standardized, limiting direct comparability. Safety outcomes such as tamponade and effusion were infrequent events, and the analysis may have been underpowered to detect modest differences. Furthermore, technological advances in ICE and fluoroscopy systems over the time span of included studies could have influenced procedural performance. Finally, publication bias cannot be fully excluded, although funnel plots did not reveal significant asymmetry.

Cost considerations were not directly evaluated in this analysis. ICE‐guided procedures typically require an additional venous access for catheter insertion and involve disposable imaging catheters, which may increase upfront procedural costs. These factors should be weighed against potential savings from reduced fluoroscopy, lower radiation exposure, and avoidance of general anesthesia in selected patients.

## 6 Clinical Implications and Future Perspectives

From a clinical perspective, ICE‐guided TSP offers a radiation‐sparing strategy without compromising procedural success or safety, supporting its broader adoption in contemporary electrophysiology and structural heart interventions. Future studies should prioritize randomized comparisons of ICE with TEE and fluoroscopy‐only approaches, incorporate standardized radiation and efficiency metrics, and evaluate newer technologies such as three‐dimensional and high‐resolution ICE. Cost‐effectiveness analyses and assessment of workflow efficiency will be particularly important to inform real‐world implementation. As operator experience increases and zero‐fluoroscopy strategies expand, ICE is likely to assume an increasingly central role in left atrial access.

## Author Contributions

**Asim Mohammed Eldai Abdalla:** writing and supervision. **Kolluru Pavani Durga:** conceptulization, methodology, writing. **Animisha Chukka:** formal analysis, data correction. **Mounika Kotte:** project administration, writing, revision. **Muhammad Awais:** writing, validation, software, investigation. **Abida Perveen:** supervision, methodology, writing. **Jahanzeb Malik:** project administration, writing, revision.

## Funding

The authors have nothing to report.

## Conflicts of Interest

The authors declare no conflicts of interest.

## Data Availability

Data sharing not applicable to this article as no datasets were generated or analysed during the current study.
